# Molecular Characterization of Multidrug-Resistant Shigella flexneri

**DOI:** 10.7759/cureus.53276

**Published:** 2024-01-31

**Authors:** Kshitij Satija, Vaibhav P Anjankar

**Affiliations:** 1 Medicine, Jawaharlal Nehru Medical College, Datta Meghe Institute of Higher Education and Research, Wardha, IND; 2 Anatomy, Jawaharlal Nehru Medical College, Datta Meghe Institute of Higher Education and Research, Wardha, IND

**Keywords:** antibiotic resistance, multidrug-resistant organism (mdro), human population genetics, multidrug resistant (mdr), shigella

## Abstract

Due to their propensity for causing diarrheal illnesses and their rising susceptibility to antimicrobials, Shigella infections constitute a serious threat to global public health. This extensive study explores the frequency, antibiotic resistance, genetic evolution, and effects of Shigella infections on vulnerable groups. The research covers a wide range of geographical areas and sheds information on how the prevalence of Shigella species is evolving. Shigella strain antimicrobial resistance patterns are thoroughly examined.

Multidrug resistance (MDR) has been found to often occur in investigations, especially when older antimicrobials are used. The improper use of antibiotics in China is blamed for the quick emergence of resistance, and variations in resistance rates have been seen across different geographical areas. Shigella strains' genetic makeup can be used to identify emerging trends and horizontal gene transfer's acquisition of resistance genes. Notably, S. sonnei exhibits the capacity to obtain resistance genes from nearby bacteria, increasing its capacity for infection. The study also emphasizes the difficulties in accurately serotyping Shigella strains due to inconsistencies between molecular and conventional serology. These results highlight the necessity of reliable diagnostic methods for monitoring Shigella infections. In conclusion, this study emphasizes how dynamic Shigella infections are, with varying patterns of occurrence, changing resistance landscapes, and genetic adaptability. In addition to tackling the rising problem of antibiotic resistance in Shigella infections, these findings are essential for guiding efforts for disease surveillance, prevention, and treatment.

## Introduction and background

Shigellosis is one of the leading causes of diarrhea-related morbidity and mortality, especially in developing nations. It is characterized by symptoms such as diarrhea and/or dysentery with frequent mucous red stools, abdominal cramps, and tenesmus [1]. Shigella, a small, unencapsulated, non-motile, Gram-negative rod, has an estimated global annual incidence of 164.7 million cases, among which 163.2 million occur in developing countries, leading to 1.1 million deaths. Moreover, almost more than half of all episodes and a majority of all Shigella-related deaths involve children younger than five years of age [2]. The three antibiotics ceftriaxone, trimethoprim-sulphamethoxazole, and ciprofloxacin are most frequently used to treat shigellosis. The identification of S. sonnei strains that produce extended-spectrum lactamases was previously described and it was proven that these strains' decreased sensitivity to fluoroquinolones contributed to the rise of quinolone-resistant Shigella in Korea [3].

Shigellosis often resolves on its own. Although severe dehydration is rare, oral rehydration is strongly advised. Additionally, depending on how severe the illness is, quinolones and cephalosporins are the preferred antibiotic treatments [4]. Utilizing molecular characterization techniques for Shigella flexneri (S. flexneri) can significantly enhance the precision of subtype identification and facilitate tracing the origins of new serotypes during pathogen outbreaks. Furthermore, this approach enables the assessment of pathogen dissemination across various geographic regions, the emergence of variants, and the identification of closely related strains within a lineage. Additionally, analyzing antibiotic resistance and resistance genes aids in comprehending drug resistance mechanisms, which in turn assists in guiding effective clinical treatment strategies for the disease. S. flexneri exhibits a minimum of 15 distinct serotypes, which can be differentiated through serological analysis. These serotypes include 1a, 1b, 1c, 2a, 2b, 3a, 3b, 4a, 4b, 5a, 5b, Y, X, Xv, and 6 [5]. In terms of its evolutionary relationships, S. flexneri exhibits two distinct lineages. One lineage is exclusively composed of S. flexneri serotype 6, while the other encompasses all the remaining serotypes. The latter lineage is further classified into seven phylogroups (PGs), which were identified through single nucleotide polymorphisms. PGs 1-3 hold a prominent position, collectively accounting for 81% of the strains. Despite certain serotypes dominating specific PGs (e.g., serotype 1b, 3a, and 2a for PG1, PG2, and PG3, respectively), it is important to note that each phylogroup includes multiple serotypes, as the PGs are not exclusively defined by serotype distinctions.

Globally, S. flexneri serotype 2a of PG3 emerges as the most prevalent strain [6]. Shigellosis typically results from drinking contaminated water from open environmental sources, although factors influencing the formation and waning of Shigella epidemics are still poorly understood. Nonetheless, a prior study indicates that these organisms may only endure for a restricted period in environmental waters. Conversely, it has been demonstrated that Shigella spp. can persist for an extended duration within the cytoplasm of free-living amoebae, leading to the hypothesis that amoebae could act as a reservoir for aquatic transmission [7].

## Review

Search methodology

The research articles included in the text offer a thorough investigation of all aspects of Shigella infections. These investigations were carried out in various places, including Pondicherry, India; Kolkata, India; Guizhou, China; Tehran, Iran; and Southeast Brazil. These studies' main goal was to learn more about Shigella infections, including their prevalence, genetic makeup, patterns of antibiotic resistance, and regional variations. The researchers used a complex technique to attain these goals. People exhibiting diarrhea and dysentery, two symptoms linked to Shigella infections, had their stool samples collected. Shigella strains were identified and categorized using a number of methods in later laboratory tests. Traditional biochemical testing, polymerase chain reaction (PCR) assays, serological examinations, and sophisticated molecular profiling techniques like pulsed-field gel electrophoresis (PFGE) were among them. Serotyping, frequently based on PCR-based techniques, was used to identify the precise serogroups and serotypes within the Shigella samples.

Key Shigella genes, including ipaH, ial, sen, set1A/B, and stx1, were found by genetic studies, assisting in the distinction of Shigella species and serotypes. The researchers also examined Shigella strains' antimicrobial resistance profiles, evaluating their sensitivity to a range of drugs often used in clinical settings. The gathering of epidemiological information, such as the frequency of various Shigella species, as well as patient demographics, such as age and gender, were also included in these investigations. To identify trends and changes in Shigella epidemiology and antimicrobial resistance, several research used temporal analyses, comparing Shigella strains and resistance patterns throughout various time periods. Shigella prevalence and antibiotic resistance regional variations were also investigated, allowing comparisons across areas and nations. These efforts culminated in a thorough analysis of the results that highlighted any potential public health concerns. These investigations brought to light the difficulties in treating Shigella infections, especially in light of the growing antibiotic resistance, and they emphasized the critical need for prudent antimicrobial usage in clinical practice. As a whole, these research projects sought to offer insightful information on the frequency, genetic variety, and antibiotic resistance of Shigella strains, providing a basis for sensible preventive and treatment methods for Shigella infections across the world. 

**Figure 1:**
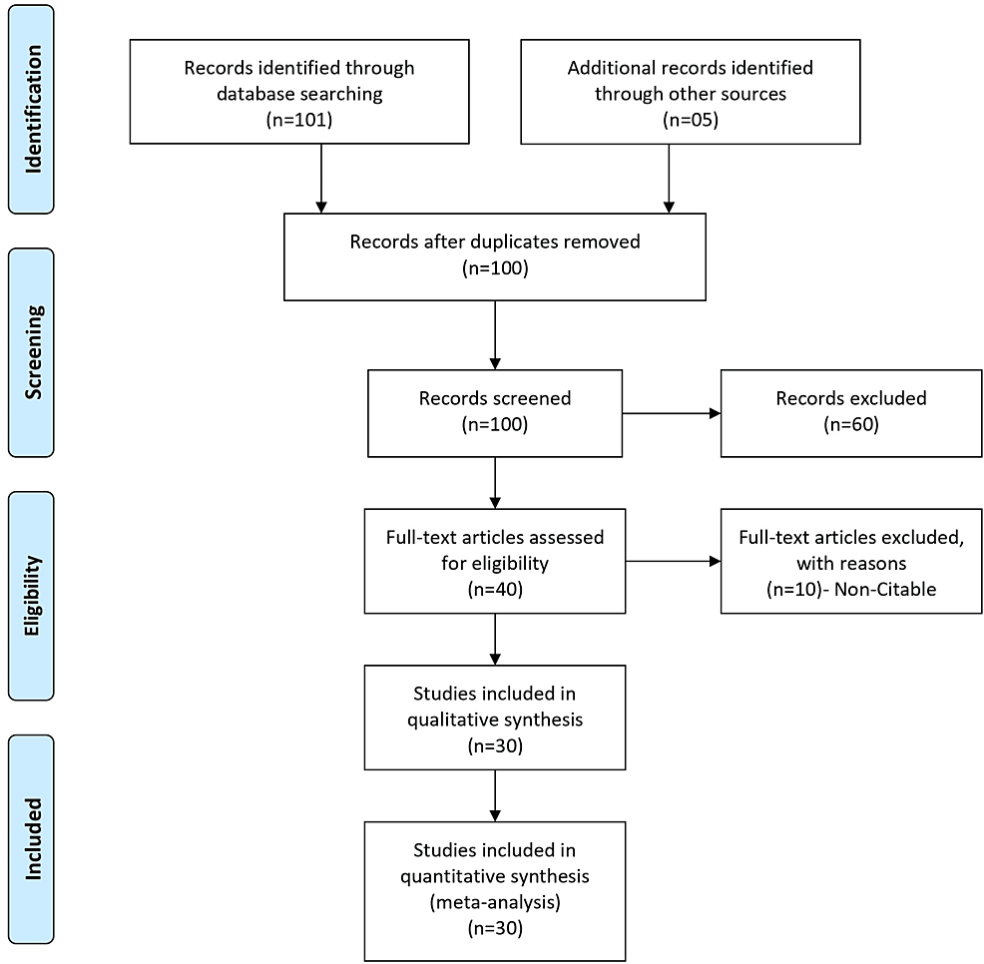
PRISMA flow diagram PRISMA: Preferred Reporting Items for Systematic Reviews and Meta-Analyses

The authors conducted a thorough literature review of shigellosis, utilizing key databases such as PubMed, MEDLINE, Scopus, and Embase. Their search strategy combined relevant keywords and controlled vocabulary to capture comprehensive information on Shigella infections. The selected databases are well-known for their coverage of biomedical and health-related literature. In addition to the initial database search, the authors likely employed citation chaining, consulted experts, and explored other relevant sources to identify five additional articles. The decision to include these articles was likely based on their significance in contributing valuable insights to the understanding of shigellosis, its prevention, treatment strategies, and public health implications on a global scale. Figure 1 explains and elaborates the search methodology.

Shigella infection and antimicrobial resistance trends

Multidrug-resistant organisms (MDROs) earn this label due to their ability to resist numerous antimicrobial agents in vitro. MDRO infections have been associated with adverse patient outcomes, potentially leading to inadequate or delayed antimicrobial therapy [8]. Diarrhea resulting from Shigella infection has traditionally been effectively treated with a five-day course of antimicrobial agents, following recommendations by the World Health Organization (WHO). However, since the 1940s, the treatment of shigellosis has become increasingly complex due to the emergence of antimicrobial resistance. The first recognized instance of sulfonamide resistance along Shigella isolates occurred in Japan during that time. Subsequently, resistance to tetracycline and chloramphenicol in Shigella strains became evident during the 1950s. As tetracycline resistance developed, ampicillin became the preferred drug for treatment. Nevertheless, ampicillin-resistant strains started to surface in the early 1980s, becoming prevalent in Asia and Africa. To address this, co-trimoxazole became the new drug of choice and remained effective until the mid-1980s when resistance to this antibiotic emerged. As a result, the susceptibility of Shigella strains to co-trimoxazole decreased significantly, as shown in Table 1.

**Table 1:** Susceptibility of co-trimoxazole to Shigella strains in different locations. Reference [8].

Year	Location	Shigella Strain	Susceptibility to Co-trimoxazole
Early 1980s	Dhaka, Bangladesh	All strains	100%
1984	Dhaka, Bangladesh	All strains	45%
2015-2016	Tehran, Iran	Shigella spp.	7.9% of all cases
2015-2016	Tehran, Iran	S. sonnei	53.33%
2015-2016	Tehran, Iran	S. flexneri	44.0%
2015-2016	Tehran, Iran	S. dysenteriae	1.33%
2015-2016	Tehran, Iran	S. boydii	1.33%
2015-2016	Tehran, Iran	Hospitalization	14.66% of Shigella cases
2015-2016	Tehran, Iran	S. sonnei (hospitalized)	17.5% of hospitalized cases
2015-2016	Tehran, Iran	S. flexneri (hospitalized)	33% of hospitalized cases
2015-2016	Tehran, Iran	S. dysenteriae (hospitalized)	1.33% of hospitalized cases
2015-2016	Tehran, Iran	Age ≤5 years (S. sonnei)	30%
2015-2016	Tehran, Iran	Age >5 years (S. sonnei)	70%
2015-2016	Tehran, Iran	Age ≤5 years (S. flexneri)	51.50%

The susceptibility of Shigella strains to co-trimoxazole in Dhaka, Bangladesh, over time and the prevalence of different Shigella species in a study conducted in Tehran, Iran, can also be taken as an example [9]. Molecular characterization of Shigella flexneri (S. flexneri) is mentioned as a method to enhance the precise identification of distinct subtypes of this Shigella species and to determine the origin of new serotypes. This molecular characterization can aid in investigating the sources of infection during outbreaks of this pathogen.

A study also examined Shigella infections in a specific region and found variations in the prevalence of different Shigella species among infected individuals [10]. Specifically, Shigella Sonnei (S. sonnei) and S. flexneri were the most common species, with S. sonnei affecting more males. The research emphasized the influence of age and gender on the distribution of these infections. Previous studies from different regions in Iran had identified different predominant serogroups, and this study categorized Shigella isolates into distinct patterns using enterobacterial repetitive intergenic consensus polymerase chain reaction (ERIC-PCR) and multilocus sequence typing (MLST). The results revealed the presence of two distinct sequence types (ST152 and ST245), with ST152 being the most prevalent. Furthermore, a separate study in Tianjin, China, observed a shift in Shigella strains over time, with changes in resistance patterns to traditional antibiotics, particularly an increase in cotrimoxazole resistance over a 26-year period.

The study highlighted the dynamic nature of Shigella infections, with varying predominant serogroups in different regions and timeframes. It underscored the importance of understanding the factors influencing the distribution of these infections, including age and gender [11]. The use of molecular typing methods like ERIC-PCR and MLST provided valuable insights into the genetic diversity of Shigella strains. Additionally, the shift in resistance patterns to traditional antibiotics over time suggested the need for ongoing monitoring and research to address the evolving challenges posed by Shigella infections. The detailed findings from the study are presented in Table 2.

**Table 2:** Shigella study. References [10-11]. Shigella strain S. sonnei was found to be the most prevalent in infected males in a study, while S. flexneri predominated in Southwest Iran. Each Shigella strain exhibited distinct ERIC patterns as a result of molecular profiling, and MLST indicated two primary sequence types. Shigella strain prevalence in the Tianjin region of China changed over time, with S. sonnei experiencing a notable rise by 2009–2010. Furthermore, from 1981 to 1983, when antibiotic resistance for some medications decreased, until 2009–2010, when cotrimoxazole resistance grew to 100%. This drop in resistance, however, was not statistically significant. Despite being informative, the results could have varied meanings depending on the study's techniques and the place it was conducted.

Attribute	1981-1983 Data	2009-2010 Data	Other Data
Shigella in Males			S. sonnei: 23 (57.5%)
-	-	-	S. flexneri: 10 (30.3%)
Dominant Serogroup (Iran)	-	-	Southwest: S. flexneri
-	-	-	Tehran: S. sonnei
ERIC-PCR Profile	-	-	S. flexneri: Yes
-	-	-	S. sonnei: Yes
-	-	-	S. boydii: Yes
Sequence Type	-	-	ST152: Most prevalent
-	-	-	ST245: Present
Tianjin Strains	Total: 30	Total: 66	-
-	S. flexneri: 30 (100%)	S. flexneri: 29 (43.94%)	S. sonnei: 36 (54.55%)
-	-	-	S. boydii: 1 (1.52%)
Resistance Patterns	Tetracycline: 76.47%-100%	Tetracycline: Reduced	-
-	Streptomycin: 76.47%-100%	Streptomycin: Reduced	-
-	Chloramphenicol: 76.47%-100%	Chloramphenicol: Reduced	-
-	Gentamicin: Not resistant	Gentamicin: Not mentioned	-
-	Cotrimoxazole: 46.70%	Cotrimoxazole: 100%	

Changing antimicrobial sensitivity patterns over time

In a comparative analysis of Shigella resistance patterns over two time periods (1981-1983 and 2009-2010), significant changes were observed. Shigella strains during the earlier period were highly sensitive to several antibiotics, including amikacin, gentamicin, third- and fourth-generation cephalosporins, quinolones, and imipenem. However, in the more recent period, there was an increasing trend of resistance, particularly for third- and fourth-generation cephalosporins and quinolones. S. flexneri displayed a more pronounced increase in resistance compared to S. sonnei, with significant differences observed, especially in the case of third- and fourth-generation cephalosporins. This evolving resistance pattern among Shigella strains highlights the dynamic nature of antibiotic resistance [12].

In a separate study conducted in Tehran, Iran, molecular analysis was used to identify and classify Shigella isolates. The study found that 70% of the isolates were S. sonnei, and 30% were S. flexneri. All the Shigella strains possessed the ipaH gene, a marker specific to the Shigella genus, with additional genetic markers specific to each species. This molecular identification approach was consistent with the results obtained through serogrouping analysis, confirming the accuracy of the methods used for species identification [13]. Additionally, a study in Kolkata, India, collected stool samples from hospitalized children with acute diarrhea between January 2001 and August 2004, as shown in Table 3.

**Table 3:** Notable findings of resistance to Shigella antibiotics. References [12-13].

Antibiotic	Sensitivity (1981-1983)	Resistance (2009-2010)	Resistance Trend	Notable Findings
Amikacin	100%	Varying levels	Increasing	Shigella flexneri and Shigella sonnei exhibited statistically significant resistance in 2009-2010.
Gentamicin	100%	Varying levels	Increasing	-
Third/Fourth Gen Cephalosporins	100%	Increasing	Increasing	Shigella flexneri showed marked and statistically significant resistance increase.
Quinolones	100%	Increasing	Increasing	-
Imipenem	100%	-	-	-

Changing trends in Shigella prevalence and antibiotic resistance

In a study involving Shigella strains in a certain region, it was found that Shigella was isolated from 7.7% of patients, with S. flexneri being the most prevalent serogroup throughout the study. However, antibiotic resistance patterns evolved over the years, with some strains exhibiting increased resistance to fluoroquinolones. Shigella dysenteriae (S. dysenteriae) type 1 and S. flexneri showed higher antimicrobial resistance compared to other serogroups. The study highlights the dynamic nature of Shigella infections and the importance of monitoring antibiotic resistance [14].

Another study examined 1280 fecal samples to identify Shigella strains, with conventional biochemical tests and PCR assays used for differentiation. Shigella species were isolated from 3.8% of the samples, with S. sonnei being the most prevalent, followed by S. flexneri and S. dysenteriae. Some of the isolated strains exhibited extended-spectrum beta-lactamases (ESBLs), indicating the presence of antibiotic resistance in the region [15]. In a different research study conducted in the Chinese region of Guizhou, various S. flexneri serotypes were identified, with changes in serotype prevalence over different time periods. The distribution of serotypes highlighted the evolving nature of Shigella strains in the region [16]. Additionally, a study in the Northwest Arkansas (NWA) region observed a shift in the prevalent Shigella serovar, with S. flexneri 2a being a significant pathogen. This study involved genomic and proteomic analyses of clinical isolates collected at different time intervals to understand the serovar turnover and changes in Shigella prevalence over the years [17]. These studies collectively emphasize the importance of ongoing research to track the dynamic nature of Shigella infections and antibiotic resistance in Table 4.

**Table 4:** Information of resistance to Shigella on ESBLs. References [14-17].

Study Information	Shigella Strain Distribution	Antibiotic Resistance (2001 vs. 2002)	Extended-Spectrum Beta-Lactamases (ESBLs)
Study 1	S. flexneri (60.6%), S. sonnei (23.8%), S. dysenteriae (9.8%), S. boydii (5.7%)	Ampicillin (50% vs. 32%), Co-trimoxazole (96% vs. 83%), Tetracycline, Nalidixic Acid	Fluoroquinolone resistance in S. dysenteriae and S. flexneri increased over the years.
Study 2	S. sonnei (85.7%), S. flexneri (10.2%), S. dysenteriae (4%), no S. boydii	-	ESBLs detected in 10.2% of Shigella isolates, distributed across different strains.
Study 3	S. flexneri (Nine serotypes, including 1a, 2a, 1b, 2b, 3a), predominance of 2a	-	-
Study 4	S. flexneri prevalence, serovars include 1b, 2a, 3a, 4a, 6	S. flexneri AA479 serovar prominent between 2007-2013	Ongoing serovar turnover observed. Genomic and proteomic analyses conducted on clinical isolates collected four years apart.

In Southeast Brazil, the genetic variations of S. sonnei strains were classified into two distinct clusters, A and B, based on molecular analysis. These clusters showed significant differences in band patterns and molecular weights, with cluster A2 containing an average of 14 bands and cluster B1 having 18. Subgroups within cluster A (A1 and A2) and cluster B (B1 and B2) exhibited varying strain compositions, indicating the diversity of S. sonnei in the region [18]. In the period between 2014 and 2015, a study revealed 338 cases of Shigella S. out of 7212 stool samples, with S. flexneri being the dominant species, followed by S. sonnei, S. dysenteriae, and S. boydii. The incidence of S. sonnei cases increased annually, especially among children aged five and below, with shifting resistance patterns to antibiotics like norfloxacin and ampicillin [19]. Another study involving 4380 children with diarrhea identified 175 confirmed Shigella cases, predominantly S. flexneri. Resistance patterns varied among different S. flexneri types, with a notable proportion exhibiting resistance to trimethoprim-sulfamethoxazole and ampicillin [20]. Additionally, in Bangladesh, the incidence of shigellosis was relatively high, with rates increasing after the age of 40 and particularly affecting children under five. Comparatively, Bangladesh had higher shigellosis rates than China, Pakistan, Indonesia, Vietnam, and Thailand, emphasizing the significant burden of shigellosis in the region [21]. Specific details about S. flexneri parameters and details are available in Table 5.

**Table 5:** Flexneri parameters and details. References [18-21]. 70.58% of patients in Pondicherry, India, experienced dysentery, while 29.42% had watery diarrhea. All dysentery cases had ipaH, but none had stx1. Dysentery cases revealed variable gene presence. An American study connected S. sonnei with Stx1, which affects malignant epithelium via Gb3 receptors, to bloody diarrhea. Antibiotic resistance varies significantly geographically in China. Several dfrA variations have been connected to an increase in trimethoprim resistance in S. flexneri after 1990. In S, a particular plasmid was discovered to be connected to a gene cluster for antibiotic resistance. PFGE: Pulsed-field gel electrophoresis

Parameter	
Location & Diagnosis details	
Location	Pondicherry, India
Dysentery cases	70.58%
Watery Diarrhea	29.42% (Rest of the patients)
Gene Presence Among Dysentery Cases	
ial	8.34%
sen	12.5%
set1A/B	4.16%
ipaH	100%
stx1	Absent
Stx1 Information	Targets Gb3 receptors affecting malignant epithelium due to Gb3 expression
Antimicrobial Resistance in China	
MDR rate	85%
Resistance to older antimicrobials	Notably High
Inappropriate antimicrobial use leading to resistance	China's outpatient
PFGE Analysis	
Yunnan S. flexneri type 1b	95% similarity (Common ancestry)
Xinjiang isolates	Diverse origins
Antibiotic Resistance Variation	
Xinjiang vs. Yunnan & Shanghai	Xinjiang less severe
Shift in Trimethoprim Resistance	Increasing dominance of the resistant genotype post-1990
dfrA variants	Eight genes
Variants linked to dfrA1 fixation	Lin-3.2, 3Xv, part of 2.2
AMR Gene Cluster & Plasmid	blaTEM-1-strAB-sul2-dfrA14 co-transferred with pRC960-1 plasmid (Found in S. flexneri Y of porcine origin)
Lab Technique Efficiency	Identifying serotypes and subserotypes
PCR vs. Traditional Serology Agreement	Complete agreement in 13 serotypes
Discrepancies in Serotyping	
PCR identified serotype 1a	As 1b
PCR identified serotype Y	As X or 2a
Serotype 5	Non-standard amplification
Dysfunctional genes in some strains	3b, 5a, 5b causing antigen function loss (Consistent with prior findings in serotypes 2 and 5)
Parameter	Details
Location & Diagnosis	Details
Location	Pondicherry, India
Dysentery cases	70.58%
Watery Diarrhea	29.42% (Rest of the patients)
Gene Presence Among Dysentery Cases	
ial	8.34%
sen	12.5%
set1A/B	4.16%
ipaH	100%
stx1	Absent
American Study on S. sonnei	Linked with Stx1 to bloody diarrhea
Stx1 Information	Targets Gb3 receptors affecting malignant epithelium due to Gb3 expression
PFGE Analysis	
Yunnan S. flexneri type 1b	95% similarity (Common ancestry)
Xinjiang isolates	Diverse origins
Antibiotic Resistance Variation	
Xinjiang vs. Yunnan & Shanghai	Xinjiang less severe
Shift in Trimethoprim Resistance	Increasing dominance of the resistant genotype post-1990
dfrA variants	Eight genes
Variants linked to dfrA1 fixation	Lin-3.2, 3Xv, part of 2.2
AMR Gene Cluster & Plasmid	blaTEM-1-strAB-sul2-dfrA14 co-transferred with pRC960-1 plasmid (Found in S. flexneri Y of porcine origin)
Lab Technique Efficiency	Identifying serotypes and subserotypes
PCR vs. Traditional Serology Agreement	Complete agreement in 13 serotypes
Discrepancies in Serotyping	
PCR identified serotype 1a	As 1b
PCR identified serotype Y	As X or 2a
Serotype 5	Non-standard amplification
Dysfunctional genes in some strains	3b, 5a, 5b causing antigen function loss (Consistent with prior findings in serotypes 2 and 5)
Parameter	Details
Location & Diagnosis	Details
Location	Pondicherry, India
Dysentery cases	70.58%
Watery Diarrhea	29.42% (Rest of the patients)

Shigella research and findings: prevalence, serotypes, antimicrobial resistance, and horizontal gene transfer

Several studies and findings related to Shigella species, their prevalence, antibiotic resistance, and genetic characteristics were discussed. In one study conducted in Pondicherry, India, it was observed that 70.58% of patients had dysentery, with the majority of cases caused by S. flexneri. Notably, the presence of Stx1 in S. sonnei was linked to bloody diarrhea and its potential impact on malignant epithelium due to Gb3 receptor expression [22]. Another study highlighted the prevalence of multidrug-resistant Shigella strains, primarily resistant to older antimicrobials, in China. It emphasized the geographic variation in antibiotic resistance, with factors like customs, climate, and economies influencing antimicrobial susceptibility [23]. Furthermore, a study in China identified a significant shift in trimethoprim resistance in S. flexneri over time, with certain variants linked to resistance and plasmid-borne antimicrobial resistance (AMR) gene clusters observed [24].

In Southern Mozambique, the prevalence of Shigella species was investigated, with S. flexneri being the most common, followed by S. sonnei [25]. The study highlighted high rates of antibiotic resistance, particularly to drugs like trimethoprim/sulfamethoxazole, tetracycline, chloramphenicol, and ampicillin, with S. flexneri accounting for the majority of resistance cases [26]. Another study in the same region reiterated the prevalence of S. flexneri and its dominant serotypes, along with the alarming antibiotic resistance observed in Shigella species [27]. Additionally, a novel strain of Shigella in the Shigella genus was identified through saccharification activity, isolated from a culture utilizing rumen fluid as a carbon source [28]. In a study conducted in the Chinese province of Henan, the prevalence of Shigella infection was found to vary between rural and urban areas, with some regions having higher rates than others, reflecting differences in investigation practices [29]. Lastly, S. sonnei was noted to acquire resistance genes from nearby bacteria, leading to its ability to develop infections and potentially outcompete susceptible bacteria, with plasmids carrying genes for colicin production/immunity and resistance to third-generation cephalosporins [30]. Table 6 shows tabulated data of antibiotic resistance to various Shigella strains for the same.

**Table 6:** Prevalence and characteristics of antibiotic resistance in various Shigella strains. References [22-30]. MDR stands for multidrug resistance, and it refers to the resistance of a microorganism, such as bacteria, to multiple antimicrobial drugs. The specific number of antibiotics to which a species of Shigella or any other bacteria is considered MDR can vary and is not strictly defined by a specific number. Generally, MDR implies resistance to at least three or more antibiotics commonly used to treat infections caused by that microorganism. The number may vary depending on the context and the guidelines set by health organizations. It is essential to note that the development of antimicrobial resistance is a dynamic process, and bacterial strains may acquire or lose resistance to different antibiotics over time.

Study Information	Shigella Strain Distribution	Antibiotic Resistance	Prevalence and Characteristics
Study 1 (Pondicherry, India)	Dysentery (70.58%), watery diarrhea	Stx1-negative, Dysentery subtypes (ial, sen, set1A/B, ipaH)	S. sonnei linked to Stx1 and cancer susceptibility.
Study 2 (China)	MDR (85%), resistance to older antibiotics	Geographic variation in antibiotic resistance	S. flexneri type 1b in Yunnan showed common ancestry.
Study 3	Shift in trimethoprim resistance, dfrA variants	Multiple dfrA variants linked to resistance	Unique AMR gene cluster identified in S. flexneri.
Study 4	PCR identification of serotypes, discrepancies	Dysfunction in some serotype genes	Agreement and discrepancies between PCR and traditional serotyping.
Study 5 (Mozambique)	Shigella species distribution, S. flexneri serotypes	Antimicrobial resistance prevalence	S. flexneri responsible for most antibiotic resistance.
Study 6	Isolation of Shigella from enrichment culture	High prevalence in rural Sui County	Variation in Shigella prevalence in urban and rural areas.
Study 7 (Vietnam)	S. sonnei clones, genetic bottleneck episodes	Transfer of resistance genes	Rapid development of dominant clones in S. sonnei.

Table 7 summarizes the articles included and referenced in the study.

**Table 7:** Articles included in the study.

Author	Year	Title	Summary
Ranjbar et al. [1]	2016	Molecular characterisation of quinolone-resistant Shigella strains isolated in Tehran, Iran.	The goal of the study was to investigate the molecular mechanisms that underlie Shigella strains' resistance to quinolones. Of the 73 isolates examined, 23 (or 31.5%) showed resistance to nalidixic acid; the most commonly resistant strain was Shigella sonnei. Furthermore, the qnrS gene was discovered to be present in four isolates (17.4%), whereas the qnrA and qnrB genes were not present in any sample. Leucine was substituted for serine due to a mutation in the gyrA gene. To have a more thorough understanding of Shigella isolates resistant to quinolones, more research must be done.
Gu et al. [2]	2012	Comparison of the prevalence and changing resistance to nalidixic acid and ciprofloxacin of Shigella between Europe-America and Asia-Africa from 1998 to 2009.	An extensive literature review has revealed a rising public health concern posed by Shigella's rising antibiotic resistance, which frequently results in treatment failure. Resistance rates to ciprofloxacin and nalidixic acid were 5.0% and 33.6%, respectively, in Asia and Africa, which were significantly higher than those in Europe and America by factors of 10.5 and 16.7. Resistance levels increased to 64.5% and 29.1% between 2007 and 2009. Interestingly, European strains of Shigella flexneri showed more resilience than Shigella sonnei. These results highlight the necessity of ongoing surveillance for Shigella antibiotic resistance to manage this growing public health issue.
Kim et al. [3]	2008	Resistance to fluoroquinolones by the combination of target site mutations and enhanced expression of genes for efflux pumps in shigella flexneri and shigella sonnei strains isolated in Korea.	Antibiotic susceptibility tests revealed that two strains of S. flexneri were resistant to fluoroquinolones. Three alterations in the GyrA gene and four substitutions in the ParC gene were found in these strains. It's interesting to note that when carbonyl cyanide-m-chlorophenylhydrazone was present, their susceptibility to fluoroquinolones improved, suggesting that energy-dependent active efflux pumps were involved in their resistance mechanism. Moreover, the organisms displayed the genes that produce efflux pumps, indicating that chromosomes partially mediated their resistance.
Pons et al. [4]	2013	Antimicrobial resistance in Shigella spp. causing traveller’s diarrhoea (1995-2010): a retrospective analysis.	The investigation examines how travelers returning from tropical regions develop antimicrobial resistance in Shigella spp., highlighting the significant impact this has on public health worldwide.
Yang et al. [5]	2016	Molecular characterisation and analysis of high-level multidrug resistance of shigella flexneri serotype 4s strains from China.	24 isolates of Shigella flexneri serotype 4s from 1,973 cases in China were analyzed, and it was discovered that they originated from multiple serotypes, of which two were dominant. In addition to showing strong resistance to third-generation cephalosporins, these isolates also showed resistance to ticarcillin, ampicillin, and tetracycline. The majority of them had a variety of antimicrobial resistance factors. With the potential to emerge as the next predominant serotype, this study emphasizes the significance of continuous surveillance and intervention to address this emerging multidrug-resistant (M.D.R.) serotype.
Nguyen et al. [6]	2023	Characterisation of shigella flexneri in northern Vietnam in 2012-2016.	Due to the widespread distribution of Shigella flexneri and Shigella sonnei worldwide, shigellosis represents a significant public health concern in developing countries. Interestingly, S. sonnei is gradually becoming more common than S. flexneri.
Rahman et al. [7]	2011	Recovery and characterisation of environmental variants of shigella flexneri from surface water in Bangladesh.	A study in a Bangladeshi freshwater lake resulted in the isolation and identification of five bacterial strains as Shigella flexneri 2b. These strains differed significantly from standard clinical strains despite displaying typical physiological traits of Shigella. They were discovered to be lacking other common Shigella-related virulence marker genes, but they did carry a megaplasmid and the ipaH virulence gene. The absence of a complete set of virulence genes suggests that these strains might represent atypical environmental Shigella variants that are non-invasive.
Magiorakos et al. [8]	2012	Multidrug-resistant, extensively drug-resistant and pan drug-resistant bacteria: an international expert proposal for interim standard definitions for acquired resistance.	The terms extensively drug-resistant (XDR), pan drug-resistant (P.D.R.), and multidrug-resistant (M.D.R.) are used in medical literature to describe resistance patterns in antimicrobial-resistant bacteria that are associated with healthcare settings. The European Centre for Disease Prevention and Control and the C.D.C. have developed these classifications to provide a common international language for acquired resistance profiles in Staphylococcus aureus, Enterococcus spp., Enterobacteriaceae, Pseudomonas aeruginosa, and Acinetobacter spp. Epidemiologically significant antimicrobial categories were established for each type of bacterium. It is crucial to remember that bacterial isolates must be tested against all or almost all of the antimicrobial agents included in the established categories to guarantee accurate classification.
Niyogi [9]	2012	Increasing antimicrobial resistance—an emerging problem in the treatment of shigellosis.	In developing countries, shigellosis plays a major role in the illness and mortality caused by diarrhea. Treatment difficulties arise from the presence of multidrug-resistant strains of illness, even though effective antibiotics can reduce the length of illness. Cephalosporins, azithromycin, and fluoroquinolones are good treatment choices, but it's important to watch for the early signs of resistance.
Dallal et al. [10]	2018	Molecular epidemiology and genetic characterization of shigella in pediatric patients in Iran.	In this study, Shigella isolates from pediatric patients in Tehran, Iran, were examined for the presence of integron types and β-lactamase genes (bla-CTX-M, bla-SHV, and blaTEM). Between May 2015 and August 2017, six hospitals provided separate samples of diarrheal stool from which 75 Shigella spp. were recovered. Significant resistance to ampicillin, trimethoprim/sulfamethoxazole, and nalidixic acid was found in the antimicrobial resistance tests. Notably, the Shigella isolates showed rising resistance patterns with increased integrons and E.S.B.L. genes, especially the frequency of blaCTX-M15.
Shokoohizadeh et al. [11]	2017	Molecular characterization of shigella spp. isolates from a pediatric hospital in Southwestern Iran.	In this study, children with diarrhea at a pediatric hospital in Ahvaz were recruited for the purpose of identifying common clones and genetic relationships among Shigella spp. Using multi-locus sequence typing (M.L.S.T.).
Cao et al. [12]	2012	Multi-locus sequence typing (MLST) and repetitive extragenic palindromic polymerase chain reaction (rep-pcr), characterization of shigella spp. over two decades in Tianjin China.	This study explores the evolutionary biology, phylogenetic relationships, antimicrobial resistance, and dominating serogroup changes of Shigella spp. in Tianjin over a 20-year period. The study, which uses REP-PCR and M.L.S.T., finds that group D Shigella had higher resistance rates in 2009 and 2010, particularly in Shigella flexneri, which showed increased resistance to several antibiotics. Furthermore, the analysis indicates that strains from 1981–1983 and 2009–2010 share DNA bands, indicating a change in the dominant serogroup.
Shahsavan et al. [13]	2016	Multi-locus sequence type analysis of shigellas pp isolates from Tehran, Iran.	The study looks at the genetic relationships and population structure of the multidrug-resistant S. sonnei and S. flexneri strains that cause shigellosis in children in Tehran, Iran who have diarrhea.
Pazhani et al. [14]	2005	Species diversity and antimicrobial resistance of shigella spp. isolated between 2001 and 2004 from hospitalized children with diarrhea in Kolkata (Calcutta), India.	193 Shigella strains from 2,489 hospitalized children with acute diarrhea were the subject of the study. The most prevalent serotype was found to be S. flexneri, which was followed by S. sonnei, S. dysenteriae, and S. boydii. Significantly, since 2002, S. flexneri 2a has dominated the serotype. Type 1 S. dysenteriae strains resistant to several antibiotics, including fluoroquinolones, reappeared in 2002. The research highlights how crucial it is to keep an eye on antimicrobial susceptibility to guarantee successful treatment.
Sabour et al. [15]	2022	Molecular detection and characterization of shigella spp. harboring extended-spectrum β-lactamase genes in children with diarrohea in northwest Iran.	The study looked into the frequency of Shigella strains that produce extended-spectrum β-lactamase (E.S.B.L.) in patients with diarrhea in northwest Iran. 1,280 children with diarrhea had their feces sampled between January 2019 and December 2020. Several Shigella species, such as Shigella sonnei, Shigella dysenteriae, Shigella flexneri, and Shigella boydii, were identified using multiplex PCR assays. The study found that ESBL-encoding genes are significantly more common in Shigella species, which poses a problem for treating dysentery and emphasizes the significance of closely monitoring antibiotic use in patients.
Li et al. [16]	2015	Genetic characterization of shigella flexneri isolates in Guizhou province, China.	This study looked at the genetic makeup of Shigella flexneri, a major cause of shigellosis in Guizhou Province, China. Twenty sequence types and nine distinct serotypes were found; four were the most common. 44 other types were also identified, with MT 1 being the most common. These results provide insight into the relationship between serotypes and genetic relationships and advance our knowledge of the changing causes of shigellosis in China.
Torrez Lamberti et al. [17]	2022	Genomic and proteomic characterization of two strains of shigella flexneri 2 isolated from infants’ stool samples in Argentina.	Two clinical isolates of Shigella flexneri 2a from children with gastroenteritis in the Northwest of Argentina were examined in this study. Finding molecular changes that could explain this intestinal pathogen's global prevalence was the goal.
Angelini et al. [18]	2009	Molecular epidemiology of shigella spp strains isolated in two different metropolitan areas of southeast Brazil.	The human pathogen responsible for shigellosis, Shigella spp., is highly contagious and varies in distribution across different regions, age groups, and the human development index. Shigellosis cases are frequently underreported in Brazil, where strains of S. flexneri and S. sonnei are common. Two species' worth of strain clusters were found in a study using pulsed-field gel electrophoresis. On the other hand, it demonstrated genotype diversity and these strains' effective adaptation to regional environmental conditions.
Anandan et al. [19]	2017	Molecular characterization of antimicrobial resistance in clinical shigella isolates during 2014 and 2015: trends in South India.	In Vellore, South India, this study looks into the prevalence and resistance patterns of Shigella species in the years 2014–2015. Globally, Shigella is a significant cause of acute diarrheal illnesses.
Khaghani et al. [20]	2014	Shigella flexneri: a three-year antimicrobial resistance monitoring of isolates in a children hospital Ahvaz, Iran.	To shed light on the high prevalence of acute gastroenteritis in developing countries, this study investigates the distribution of Shigella serogroups and serotypes as well as their profiles of antibiotic resistance.
Von Seidlein et al. [21]	2006	A multicentre study of shigella diarrhoea in six Asian countries: disease burden, clinical manifestations, and microbiology.	To learn more about the current disease burden, clinical manifestations, and microbiological aspects of shigellosis, a prospective study was carried out in six Asian countries. Every year, this diarrheal illness claims the lives of over a million people.
Das et al. [22]	2016	The emergence of quinolone resistant shigella sonnei, Pondicherry, India.	There has been an alarming increase in ciprofloxacin resistance in 34 Shigella sonnei isolates studied between 2012 and 2015. Minimum inhibitory concentrations (M.I.C.s) for ciprofloxacin, ofloxacin, and levofloxacin ranged from 4 to 64 for 16 of the 34 quinolone-resistant isolates among them. The study found that gyrA, gyrB, parC, and parE were among the genes with common mutations, with qnrB being the most common. Furthermore, nine of the fifteen isolates resistant to ciprofloxacin showed evidence of efflux pump activity. Understanding the quinolone resistance in these isolates better depends on these findings.
Cui et al. [23]	2015	Antimicrobial resistance of shigella flexneri Serotype 1b Isolates in China.	85% of the 40 S. flexneri 1b isolates examined in a Chinese study were multidrug resistant. Each region had different levels of antibiotic susceptibility; strains from Yunnan were more resistant than those from Xinjiang. Cephalosporin resistance was present in 15 isolates, and ampC and E.S.B.L. genes were found in some isolates, mainly from Xinjiang. The study suggests limiting the use of antibiotics to severe cases and conducting early antibiotic susceptibility testing during outbreaks. These results offer important new information for creating efficient treatment plans for S. flexneri 1b infections in China.
Chung et al. [24]	2021	Evolutionary histories and antimicrobial resistance in shigella flexneri and Shigella sonnei in Southeast Asia.	S. flexneri and S. sonnei are members of a genetically diverse genus that most likely arrived in the region between the 1970s and 1990s, according to a thorough three-decade study that looked at 1,804 whole genome sequences of Shigella from four countries in Southeast Asia. Shigella's evolution in Southeast Asia has been greatly influenced by its ability to adapt to antimicrobial pressure, as evidenced by the epidemiology of these serotypes, which are marked by recurrent clonal replacement events and evolving susceptibility to antimicrobial agents.
Brengi et al. [25]	2019	PCR-based method for shigella flexneri serotyping: international multicenter validation	Seven labs investigated a multiplex-PCR-based technique for serotyping Shigella flexneri, a common cause of human diarrhea, in an international multicenter validation study. For 14 of the 19 serotypes examined, the study found that laboratories had strong agreement, and for the remaining five serotypes, there was good alignment with conventional methods. It was determined that this PCR method was more dependable, effective, and appropriate for everyday laboratory use. As a result, the study suggests using this approach for worldwide Shigella surveillance in lab environments.
Vubil et al. [26]	2018	Antibiotic resistance and molecular characterization of shigella isolates recovered from children aged less than 5 years in Manhiça, Southern Mozambique.	The objective of this research was to evaluate the molecular epidemiology of Shigella isolates and antibiotic resistance in a case-control study conducted in southern Mozambique. The most common species found were S. flexneri, which had high antimicrobial resistance to ampicillin, tetracycline, chloramphenicol, and trimethoprim-sulfamethoxazole. There was an 8.1% case fatality rate, and 55.2% of the isolates had multi-drug resistance (M.D.R.). 22 unique clones were found in the study, with P1, P9, and P2 being the most common.
C P et al. [27]	2017	Molecular evaluation of high fluoroquinolone resistant genes in endemic cases of shigellosis, northeast part of Karnataka, India	This study investigates the increasing resistance in northern Karnataka, India, to the gastrointestinal infection Shigellosis. In particular, it looks at decreased minimal inhibitory concentrations and the diversity and molecular mechanisms of restriction endonucleases.
Wang et al. [28]	2011	Isolation and characterization of shigella flexneri g3, capable of effective cellulosic saccharification under mesophilic conditions.	Shigella flexneri G3, a novel strain of the bacteria, has been identified and investigated for its strong cellulolytic activity, especially in mesophilic and anaerobic conditions. This short, rod-shaped, gram-negative bacterium shows good cellulose conversion to glucose, cellobiose, and oligosaccharides. These features highlight its possible use in the mesophilic biofuel production process.
Xia et al. [29].	2011	Prevalence and characterization of human shigella infections in Henan province, China, in 2006.	A total of 3,531 fecal samples from patients with diarrhea were collected in Henan Province, China in 2006; 467 (13.2%) Shigella strains were isolated and serotyped. Among these, MIC analysis, pulsed-field gel electrophoresis (PFGE), and the identification of cephalosporin resistance genes were used to characterize 71 strains of Shigella flexneri. S. flexneri variant X (27.6%), S. sonnei (24.2%), and S. flexneri 2a (20.8%) were the main agents responsible for the illness. The study showed that shigellosis is quite common, with regional and gender-related variations. High resistance was seen, including total resistance to extended-spectrum cephalosporins and ciprofloxacin.
Thompson et al. [30]	2015	The rising dominance of Shigella sonnei: an intercontinental shift in the etiology of bacillary dysentery.	Shigella sonnei, a major global cause of dysentery, is expanding at an unprecedented rate in industrializing regions of Asia, Latin America, and the Middle East. This is known as shigellosis. Three important environmental factors—localized antimicrobial use exerting selective pressure, effective phagocytosis by Acanthamoeba castellanii, and natural passive immunization—may be responsible for this surge's causes. However, they are not yet fully understood. S. sonnei has an advantage over other bacteria due to its ability to obtain antimicrobial resistance genes from different bacteria. Creating a potent vaccine to prevent S. sonnei is desperately needed to fight this gastrointestinal infection that is becoming more and more common.

## Conclusions

In conclusion, the research articles offered here offer a thorough review of the frequency, trends in antibiotic resistance, and genetic differences of Shigella strains in different geographical areas and historical eras. These studies provide insight into how Shigella infections change over time and the difficulties that MDROs provide. The dynamic nature of Shigella infections is a significant finding from these investigations. Antimicrobial medicines could previously be used to treat Shigella infections, but over time, the evolution of antibiotic resistance has hindered treatment plans. High resistance rates in some areas, which result from the emergence of MDROs, highlight the urgent need for efficient surveillance and antibiotic stewardship. Shigella strains' genetic diversity and evolution have been well understood using methods like PCR and PFGE for genetic characterization. There are several serotypes and subserotypes, and some strains have genetic differences that affect the levels of virulence and antibiotic resistance. Shigella prevalence and resistance patterns vary geographically, highlighting the intricate interactions between variables such as regional socioeconomic situations, climatic conditions, and healthcare practices. The findings also stress the significance of using antibiotics correctly to prevent the spread of resistance. Overall, our results highlight the significance of ongoing study and attention in tracking Shigella infections. This study highlights the need to combat the threat that Shigella strains represent to world health and the need for novel treatment methods, vaccinations, and public health initiatives. 
